# CBCT Volumetric Changes in Combined Nasal Cavity and Paranasal Sinuses Following RAMPA-ROA Therapy: A Retrospective Cohort Study with Reference to Longitudinal Growth Data

**DOI:** 10.3390/jcm15072605

**Published:** 2026-03-29

**Authors:** Yasushi Mitani, Yuko Okai-Kojima, Mohammad Moshfeghi, Tonogi Morio, Shouhei Ogisawa, Bumkyoo Choi

**Affiliations:** 1 Codomo Clinic, Tokyo 180-0004, Japan; mitani@trust.ocn.ne.jp; 2 Children and Women Dental Clinic, Tokyo 106-0046, Japan; yukoyukono@gmail.com; 3 Department of Mechanical Engineering, Sogang University, Seoul 04107, Republic of Korea; mmoshfeghi@sogang.ac.kr; 4 Department of Oral & Maxillofacial Surgery, School of Dentistry, Nihon University, Tokyo 101-8310, Japan; tonogim@gmail.com (T.M.); ogisawa.shouhei@nihon-u.ac.jp (S.O.)

**Keywords:** RAMPA therapy, sinonasal complex, paranasal sinuses, CBCT, volumetric analysis, growth acceleration, catch-up growth

## Abstract

**Background:** The interrelationship between craniofacial morphology and respiratory function is a central focus of orthodontic and dentofacial orthopedic research. This study aimed to evaluate the volumetric changes in the sinonasal complex (combined nasal cavity and paranasal sinuses) following Right Angle Maxillary Protraction Appliance (RAMPA) therapy using cone-beam computed tomography (CBCT) and to compare these outcomes with established longitudinal growth benchmarks. **Methods:** A retrospective cohort analysis was conducted on 60 pediatric patients (24 males, 36 females; mean age: 86.60 ± 24.22 months) with radiologically clear paranasal sinuses at baseline (T1). Participants underwent RAMPA therapy for an average of 8.38 months. Volumetric quantification of the entire sinonasal complex—including the nasal cavity and all four paranasal sinuses (maxillary, ethmoid, sphenoid, and frontal)—was performed to ensure methodological alignment with existing normative growth data. **Results:** Total sinonasal volume increased significantly from 27,741.63 ± 10,675.85 mm^3^ at T1 to 32,248.00 ± 10,084.07 mm^3^ at T2 (*p* < 0.001), representing a mean gain of 4506.37 mm^3^ (16.24%). Notably, the annualized growth velocity under RAMPA therapy (6453 mm^3^/year) exceeded the physiological increment of age-matched normative data (~5418 mm^3^/year) by approximately 1.2 times. Despite a constricted baseline at T1 compared to normative values, the treatment group demonstrated a rapid “catch-up” growth trajectory. **Conclusions:** RAMPA therapy induces rapid and significant volumetric expansion of the sinonasal complex in pediatric patients, demonstrating a potent “acceleration effect” that surpasses natural physiological maturation. These findings suggest that orthopedic midfacial remodeling can effectively restructure the upper respiratory environment, bridging the gap between pathological constriction and normative developmental benchmarks in patients with maxillary hypoplasia.

## 1. Introduction

The interrelationship between craniofacial morphology, upper airway patency, and respiratory function has long been a central focus of orthodontic and dentofacial orthopedic research. Observational and imaging-based studies have demonstrated significant associations between nasal and pharyngeal airway adequacy, head posture, and craniofacial morphology in growing individuals, indicating that variations in airway dimensions are linked to characteristic skeletal and postural adaptations of the craniofacial complex [1,2,3,4]. Three-dimensional imaging investigations have further clarified that reduced airway dimensions are commonly associated with altered mandibular position and craniofacial imbalance, reinforcing the importance of airway considerations in orthodontic diagnosis and treatment planning [5,6].

Among the anatomical contributors to airway compromise, sinonasal pathology plays a critical and clinically relevant role. The radiological opacification of the paranasal sinuses typically reflects inflammatory conditions such as acute or chronic rhinosinusitis, mucosal edema, or mucus retention rather than fixed skeletal obstruction [7,8,9]. In pediatric populations, chronic rhinosinusitis is characterized by persistent inflammation of the nasal and sinus mucosa, leading to obstruction of normal drainage pathways, impaired mucociliary clearance, and reduced aeration of the sinus cavities [10,11]. Consensus guidelines and clinical reviews consistently report that such inflammatory changes reduce the effective air-filled volume of the nasal cavity and increase nasal airway resistance, thereby contributing to functional nasal airway limitation [8,11,12]. Because these inflammatory changes are potentially reversible, they introduce a dynamic component to airway obstruction that may interact with orthopedic interventions targeting the craniofacial skeleton.

Advances in three-dimensional imaging, particularly cone-beam computed tomography (CBCT), have enabled objective volumetric assessment of the sinonasal complex, including the nasal cavity and paranasal sinuses, facilitating more precise evaluation of treatment-induced structural changes [13,14]. Using these modalities, rapid maxillary expansion (RME) has been extensively investigated as a conventional orthopedic approach for correcting transverse maxillary deficiency in growing patients. Husson et al. [15] demonstrated three-dimensional oropharyngeal airway changes after facemask protraction using low-dose CT, providing a directly comparable imaging-based precedent for protraction-related airway remodeling. This strengthens the literature context for protraction effects on airway volume and supports methodological parallels for the current investigation.

A systematic review and meta-analysis demonstrated statistically significant increases in upper airway volume following RME, confirming that transverse maxillary expansion can positively influence airway dimensions [12]. Additional clinical and computational studies have shown that RME is associated with reductions in nasal airway resistance and improvements in nasal airflow, even when geometric changes in minimal cross-sectional area are modest [13,14]. These findings indicate that skeletal expansion may yield functional respiratory benefits beyond simple dimensional enlargement.

Despite these documented effects, conventional maxillary expansion and protraction approaches have limitations in controlling the direction of skeletal displacement and its secondary effects on mandibular posture. To address these limitations, alternative orthopedic strategies have been developed with the aim of producing more favorable craniofacial and airway responses.

In this context, the Right Angle Maxillary Protraction Appliance (RAMPA) system was introduced as a biomechanical evolution in maxillary orthopedic therapy. Utilizing a controlled Semi-Rapid Maxillary Expansion (sRME) protocol via an Oral Appliance (ROA), the system’s engineered capacity to deliver precise anterosuperior force vectors has been validated through multiple Finite Element Analyses (FEA) and documented clinical mechanistic studies [16,17,18]. FEA investigations demonstrate that this force system facilitates forward and upward translation of the entire maxillo-cranial complex, while clinical outcomes confirm a distinct orthopedic redistribution of stresses across circummaxillary sutures that minimizes undesirable dental side effects [19].

Beyond skeletal remodeling, the validation of RAMPA’s engineered biomechanics extends to volumetric sinonasal dimensions. FEA and clinical volumetric studies substantiated that the achieved transverse and anterosuperior displacement of the maxilla significantly increases nasal cavity dimensions and reduces airway resistance [20,21,22,23], establishing an anatomical foundation for enhanced airflow. Computational fluid dynamics (CFD) further indicate that altered airflow mechanics from orthopedic enlargement can positively influence sinonasal ventilation and exchange [24,25].

While the effects of RME, functional orthopedic appliances, and surgically assisted expansion on airway volume have been widely reported across pediatric and adult populations [26,27,28,29,30], the volumetric sinonasal effects of RAMPA therapy have been investigated primarily in the context of skeletal and biomechanical outcomes. Moreover, few studies have examined how baseline sinonasal inflammatory status may influence airway response to orthopedic maxillary protraction. Given that sinus opacification represents a potentially reversible soft-tissue obstruction rather than a fixed anatomical constraint, its interaction with anterosuperior maxillary protraction warrants systematic evaluation.

The purpose of this retrospective cohort study was to evaluate the volumetric changes in the combined paranasal sinuses and nasal cavity compartments following RAMPA-ROA therapy using CBCT. Furthermore, this study sought to perform an exploratory assessment of these clinical outcomes in reference to established natural growth baselines from the existing literature [31]. By examining the magnitude of change relative to reported physiological maturation of the sinonasal complex, we aimed to clarify the potential role of this orthopedic intervention in restructuring the maxillofacial complex and its associated nasal and sinus spaces in pediatric patients.

## 2. Materials and Methods

### 2.1. Study Design and Ethical Considerations

The investigation employed a retrospective comparative cohort study design. This approach involved analyzing existing data from two distinct groups of pediatric patients who had previously undergone RAMPA therapy. Ethical approval for this retrospective analysis was obtained from the Ethics Committee of Nihon University School of Dentistry in Tokyo, Japan (EP22D001). All procedures were conducted in accordance with the informed consent statements obtained from the parents or legal guardians of the pediatric participants at the time of clinical treatment, authorizing the use of their records for research purposes.

**Figure 1:**
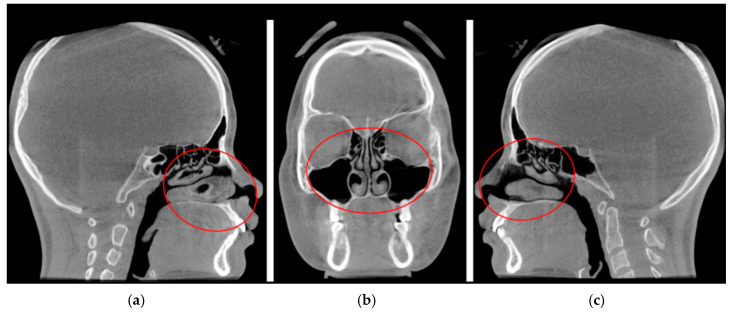
Pre-treatment CBCT assessment of paranasal sinus patency. Representative images show clear paranasal sinuses and patent sinus ostia (red circles). The absence of mucosal thickening ensures an accurate baseline for subsequent volumetric quantification in the (**a**) right sagittal, (**b**) coronal, and (**c**) left sagittal planes.

Inclusion criteria for this group stipulated that patients presented with conditions deemed suitable for RAMPA therapy (such as unilateral or bilateral posterior crossbite or other orthodontic/craniofacial reasons) and, crucially, exhibited radiologically clear paranasal sinuses on their baseline cone-beam computed tomography (CBCT) scans. “Clear” was defined as the absence of significant mucosal thickening, fluid retention, or polypoid changes that could appreciably compromise baseline airway volume due to sinus pathology, opening the sinus ostium (Figure 1).

### 2.2. Participant Selection

A total of 60 pediatric patients (24 males, 36 females) were included in this study, with a mean age of 86.60 ± 24.22 months at the start of treatment. Inclusion criteria focused on patients requiring orthopedic intervention for maxillary hypoplasia who had complete pre-treatment (T1) and post-treatment (T2) 3D imaging records. The average treatment duration was 8.38 ± 3.88 months, and the baseline sinonasal volume (T1) was 27,741.6 ± 10,675.9 mm^3^. The detailed baseline demographic and clinical characteristics of the study population are summarized in Table 1. To ensure effective orthopedic remodeling and achieve optimal therapeutic outcomes, patients were instructed to wear the RAMPA for a minimum of 12 h per day. Compliance was monitored through daily wear logs completed by the parents or guardians, which were verified by the clinician at each monthly visit. Only patients who demonstrated consistent adherence to the prescribed wear time were included in this analysis to ensure the reliability of the treatment outcomes.

### 2.3. Orthopedic Appliance and Clinical Protocol

The Right Angle Maxillary Protraction Appliance (RAMPA) system is a specialized orthopedic and orthodontic treatment modality engineered to correct specific craniofacial discrepancies. This integrated system consists of a customized extraoral framework that delivers precise forces for the anterosuperior protraction of the maxillo-cranial complex [16,17,18], and an intraoral appliance, known as the RAMPA Oral Appliance (ROA). The ROA facilitates Semi-Rapid Maxillary Expansion (sRME), typically activated by the patient at home at a rate of one quarter-turn (0.25 mm) per day. Compared to conventional Rapid Maxillary Expansion (RME), this sRME protocol aims to apply continuous, physiologic orthopedic forces over a longer duration.

**Figure 2:**
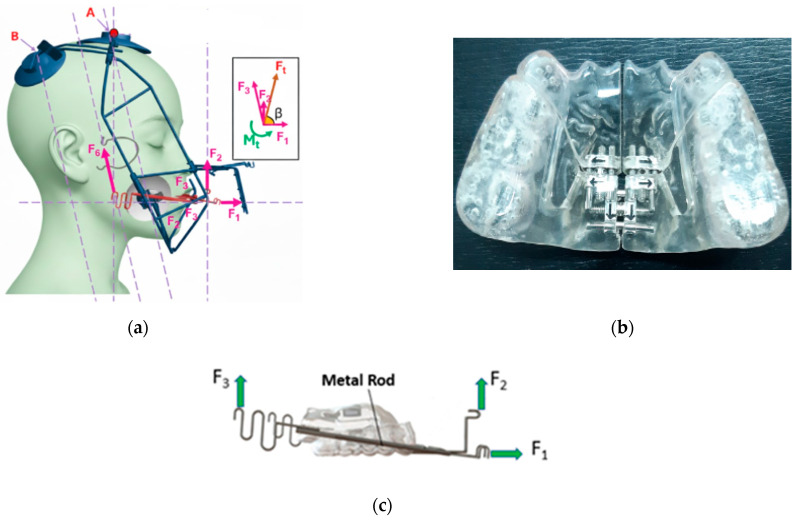
Components of the RAMPA system for craniofacial orthopedic treatment. (**a**) The extraoral RAMPA device designed for skeletal protraction, (**b**) the RAMPA Oral Appliance (ROA) featuring expansion screws, and (**c**) the metallic bow assembly that connects the ROA to the extraoral frame to transmit orthopedic forces (F_1_, F_2_, F_3_).

Unlike traditional orthopedic appliances, the RAMPA system is engineered to apply three distinct force vectors (F_1_, F_2_, F_3_) to achieve a “boosting effect” against gravity (Figure 2): the system is strategically engineered to apply directed force vectors designed to pull the maxilla in a combined anterior (forward) and superior (upward) trajectory. This biomechanical action is intended to induce a forward rotation of the palatal plane (the ANS-PNS line), thereby encouraging a favorable forward and horizontal growth pattern of the mandible. The efficacy of the system relies on three distinct force vectors: F_1_ (Horizontal Antero-superior) initiates the forward and upward movement of the midpalatal plane, F_2_ (Purely Upward) delivers an upward force designed to counteract the undesirable extrusion of anterior teeth and stabilize the vertical position, and F_3_ (Forward-Rotating) serves as the primary driver for the forward rotation of the palatal plane.

These forces are typically applied symmetrically through the use of orthopedic elastic bands. This force system facilitates a counterclockwise (CCW) rotation of the mandible, which is theorized to enlarge the retroglossal and total airway space. Clinical efficacy required consistent wear exceeding 12 h per day, with a mean treatment duration of 8.38 months.

### 2.4. Data Acquisition and Volumetric Analysis

Nasal cavity and paranasal sinuses volumes were quantified using Cone-Beam Computed Tomography (CBCT) scans acquired at T1 and T2. To ensure objective quantification, the DICOM files were analyzed using 3D imaging software (Mimics Innovation Suite; Materialise version 27.0, N.V., Leuven, Belgium). The air-filled spaces were segmented using a calibrated threshold range of −1000 to −625 HU. This setting was validated by both previous studies for pediatric airway analysis and our own pilot study, which included internal sampling of air space pixel intensities to accurately delineate the interface between respiratory cavities and surrounding soft tissues. These parameters were specifically optimized for the Alphard-3030 system (80 kVp, 5 mA, 0.3 mm voxel size) with a field of view (FOV) encompassing the entire craniofacial complex from the frontal sinus to the hyoid bone.

To enhance the accuracy of the volumetric quantification and minimize CBCT-related scattering, a Gaussian Noise Reduction filter was applied prior to segmentation. Notably, while the initial segmentation was automated, the author conducted detailed manual refinement for each case. Particular attention was paid to anatomically intricate areas, such as the nasal meatus and the paranasal sinus ostia, to ensure that these narrow passages were accurately delineated and not prematurely closed by automated thresholding. Furthermore, a Laplacian Smoothing algorithm was employed during 3D reconstruction to refine the segmented boundaries and reduce artifacts. The primary outcome measure was the combined volumetric data, calculated as the bilateral sums of the paranasal sinuses and the nasal cavity. To ensure methodological alignment with the reference growth data [31], the volumetric analysis was confined to the sinonasal complex (from the external nares to the choanae); lower pharyngeal compartments, such as the nasopharynx, oropharynx, and retroglossal spaces, were excluded from this study.

The volumetric analysis focused on the paranasal sinuses and the nasal cavity. Our measurement of the nasal cavity followed the same anatomical landmarks as Yamakawa et al. [31], defined from the external nares to the choanae. By excluding the lower pharyngeal compartments and strictly adhering to these boundaries, we ensured that our data are anatomically equivalent and directly comparable to the established longitudinal growth baselines.

**Figure 3:**
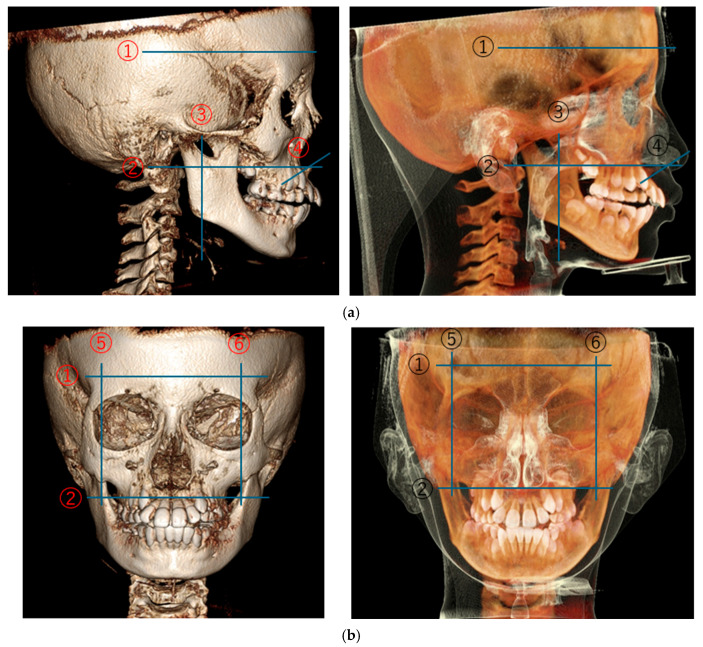
Defined anatomical boundaries for volumetric analysis of the paranasal sinuses and nasal cavity. (**a**) Sagittal and (**b**) coronal views illustrating the reference planes: ① superior border of the frontal sinus, ② floor of the nasal cavity, ③ anterior border of the adenoids, ④ external nares, and ⑤, ⑥ maximum lateral prominence of the paranasal sinuses.

The primary outcome measure was the combined volumetric data, defined as the sum of the bilateral paranasal sinuses and the nasal cavity (from external nares to choanae). To ensure anatomical consistency, the measurement boundaries extended from the anterior edge of the adenoids to the external nostrils. All volumetric data presented in the results represent these combined anatomical spaces to allow for a direct comparison with established longitudinal growth baselines (Figure 3).

To ensure reproducibility, patients were scanned in a standardized seated upright position with the Frankfort horizontal plane parallel to the floor. All participants were instructed to maintain a resting tongue position and to hold their breath at the end-expiration phase to minimize volumetric errors induced by respiratory movement. While this combined approach captures the total expansion of the paranasal and respiratory complex, it is important to note that physiological airway growth in this age group is typically slow and linear [32]. Therefore, the observed rapid volumetric changes are predominantly interpreted as a reflection of the orthopedic remodeling induced by the RAMPA-ROA system.

To ensure the technical rigor and objectivity of this quantification, 20% of the total sample (*n* = 12) was randomly selected for reliability analysis. These cases were re-analyzed by the primary investigator and an additional calibrated observer after a two-week interval to eliminate memory bias. Intraclass Correlation Coefficient (ICC) values for both intra- and inter-observer reliability were found to be above 0.90, confirming excellent reproducibility of the landmark identification and the manual refinement-inclusive segmentation protocol.

**Figure 4:**
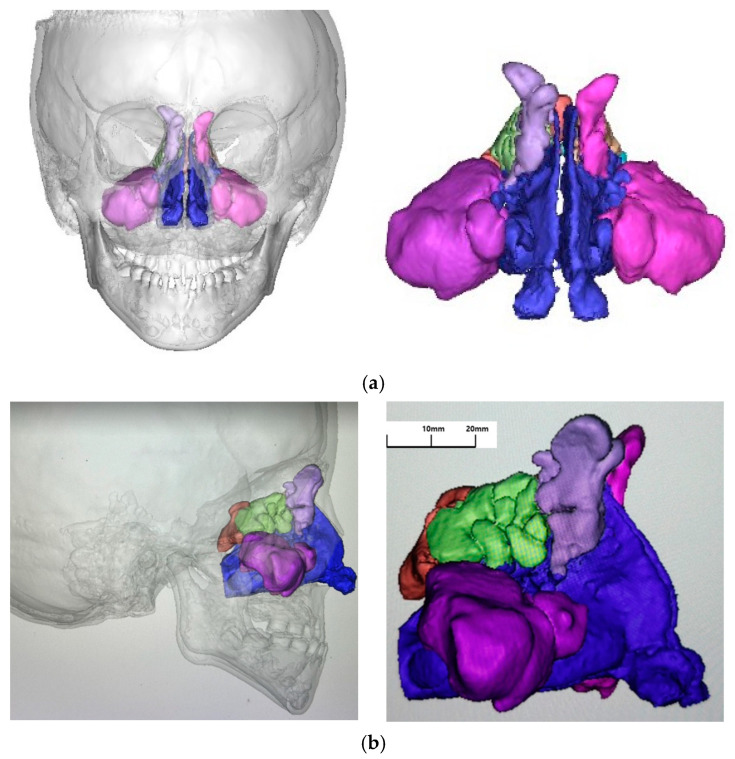
Representative three-dimensional reconstruction and segmentation of the sinonasal complex. (**a**) Coronal and (**b**) sagittal views illustrating the volumetric quantification. The sinonasal compartments are distinctively color-coded to demonstrate target anatomical boundaries: blue represents the nasal cavity, purple the maxillary sinus, light green the ethmoid sinus, light purple the frontal sinus, and brown the sphenoid sinus. Scale bars are provided as an anatomical reference to allow for an estimation of actual dimensions. Note: Pharyngeal airway spaces are visualized as translucent white but are intentionally excluded from the measurements to ensure parity with the reference study.

Volumetric measurements of the combined paranasal sinuses and nasal cavity were performed using CBCT scans acquired at two time points: baseline (T1), prior to the initiation of RAMPA-ROA therapy, and post-treatment (T2), following the completion of active therapy. Volumetric analysis was conducted using a complementary dual-software protocol to maximize analytical precision. Initially, Invivo Ver.5 (Anatomage Inc., San Jose, CA, USA) was utilized for 3D orientation and anatomical landmark identification due to its specialized orthodontic diagnostic interface. Using this software, the patient’s head position was standardized by aligning the Frankfort Horizontal (FH) plane parallel to the floor. The midline was determined by connecting the center of the nasal bone and the anterior nasal spine, and a reference line was established by connecting the mental spine of the mandible to both mandibular condyles at a right angle (Figure 3). Subsequently, the oriented data were imported into the Mimics Innovation Suite (Mimics Innovation Suite; Materialise version 27.0, N.V., Belgium) for threshold-based segmentation and precise volumetric quantification, leveraging its advanced engineering capabilities for complex 3D modeling. The air-filled spaces were segmented using a calibrated threshold range of −1000 to −625 HU. The anatomical boundaries for the measurement extended from the anterior edge of the adenoid to the external nostril (Figure 4). Nasal cavity and sinus volumes were calculated in cubic millimeters (mm^3^) for T1 and T2 images separately. To ensure consistency, all landmark identifications and measurements were performed by the same investigator.

### 2.5. Natural Growth Benchmarking

To distinguish therapeutic effects from natural ontogenetic changes, the results were compared against natural development baselines derived from established longitudinal data [31].

The natural growth benchmarks included median volumes for the paranasal sinuses and nasal cavity for age groups ranging from 3 to 24 years. In addition, growth completion trajectories indicating that the nasal cavity continues to expand until age 22, typically lagging 2–4 years behind general height growth.

### 2.6. Comparative Analysis with Natural Growth Baselines

To distinguish the therapeutic effects of RAMPA from inherent physiological maturation, the results were compared against established natural development baselines.

- Reference Data: Natural growth trajectories were derived from recent longitudinal studies [31], which provide volumetric standards for the nasal cavity and paranasal sinuses from ages 0 to 24.
- Growth Velocity Comparison: The observed volumetric increase during the 8.38-month treatment period was compared against the median annual growth rates of untreated children in the same age bracket (approx. 7–8 years).
- Acceleration Factor: An “acceleration factor” was calculated to determine how many years of natural growth were achieved within the shortened timeframe of active RAMPA therapy.

### 2.7. Statistical Analysis

To ensure the robustness of the statistical analysis, only patients with complete pre-treatment (T1) and post-treatment (T2) CBCT records were included in the final cohort. Case-wise deletion was applied to any records with significant motion artifacts or incomplete anatomical coverage during the initial screening phase. As a result, the final dataset of 60 participants contained no missing values for the primary outcome measures; therefore, no imputation methods were required for statistical processing.

Statistical analysis was performed using R software (version 4.3.3; R Foundation for Statistical Computing, Vienna, Austria). Data normality was assessed for the entire sample and gender-specific subgroups using the Shapiro–Wilk test. The results confirmed a normal distribution (e.g., *p* = 0.195 for the male subgroup, N = 24), justifying the use of parametric tests. Descriptive statistics are presented as means with standard deviations (SD) and 95% confidence intervals (CIs).

To evaluate the treatment effect while controlling for potential confounders, an Analysis of Covariance (ANCOVA) was performed with post-treatment volume (T2) as the dependent variable, while pre-treatment volume (T1), age (in months), and treatment duration were included as covariates. Additionally, a multivariable linear regression model was constructed to determine the independent influence of age, sex, and treatment duration on the volumetric change (∆V).

For primary comparisons between T1 and T2, paired t-tests were utilized for the total cohort and subgroups. To mitigate Type I error inflation during multiple subgroup comparisons, *p*-values were adjusted using the Bonferroni method. Exact *p*-values are reported for all tests, with the threshold for statistical significance set at *p* < 0.05 (with highly significant results noted at *p* < 0.001).

### 2.8. Intended Effects on Craniofacial Structures and Airway

The biomechanical actions of the RAMPA system are intended to produce a cascade of effects on the craniofacial structures, which in turn are hypothesized to positively influence upper airway dimensions. Maxillary expansion, achieved by the intraoral component, serves to widen the maxilla transversely. This widening directly increases the width of the nasal cavity floor, as the palatal bones form the floor of the nasal cavity [13,14]. The anterosuperior movement of the entire maxillary complex, driven by the extraoral appliance, is expected to reposition the maxilla and associated soft tissues, including the palate and surrounding musculature. This repositioning can potentially lead to an enlargement of the sinonasal complex, specifically enhancing the volumetric capacity of the nasal passages and the associated paranasal sinuses [13,14,16]. Such structural remodeling has been shown to vary depending on circummaxillary suture stiffness [19]. The study under review posits that these skeletal alterations induce corresponding adaptations in the soft tissues, culminating in an overall increase in airway volume, an effect particularly anticipated in the clear sinus group. The importance of assessing upper airway dimensions is underscored by the understanding that increased airway resistance, often associated with constricted airways, can contribute to abnormal craniofacial growth patterns and potentially to conditions like sleep-disordered breathing [27,28].

The anterosuperior vector of maxillary movement promoted by RAMPA is a distinguishing feature. This contrasts with some other maxillary protraction appliances that may emphasize a more purely anterior or anteroinferior force [17]. The inclusion of a significant superior component in the protraction force could have specific implications for the paranasal sinuses and their drainage pathways. The maxillary sinuses, for instance, are located superior and posterior to the palate and maxillary dentition.

The anterosuperior vector of maxillary movement promoted by the RAMPA system is hypothesized to alter the spatial orientation of the sinus ostia, particularly within the ostiomeatal complex [20]. While this biomechanical consideration offers a plausible mechanistic link between appliance design and clinical observations, the actual improvement in sinus drainage and aeration remains a theoretical proposition that warrants further functional validation through dynamic imaging or rhinological assessment.

### 2.9. Disclosure of Generative AI in the Writing Process

Generative AI (Gemini 3 Flash, Google) was utilized during the revision process of this manuscript to assist in English language polishing and to ensure the logical flow of the revised text. The AI tool was used specifically for grammatical refinement and sentence restructuring based on the authors’ original scientific content. The authors reviewed and edited the AI-generated suggestions and take full responsibility for the final content of the manuscript. 

## 3. Results

### 3.1. Baseline Characteristics of the Participants

A total of 60 pediatric patients (24 males and 36 females) who completed the RAMPA-ROA treatment protocol were included in this study. The mean age at the start of treatment (T1) was 86.60 ± 24.22 months, and the mean treatment duration was 8.38 ± 3.88 months. All participants were in the mixed or early permanent dentition stage and presented with radiographically clear paranasal sinuses at baseline. The detailed demographic and clinical characteristics are presented in Table 2.

### 3.2. Volumetric Analysis of Nasal Cavity and Paranasal Sinuses

**Figure 5:**
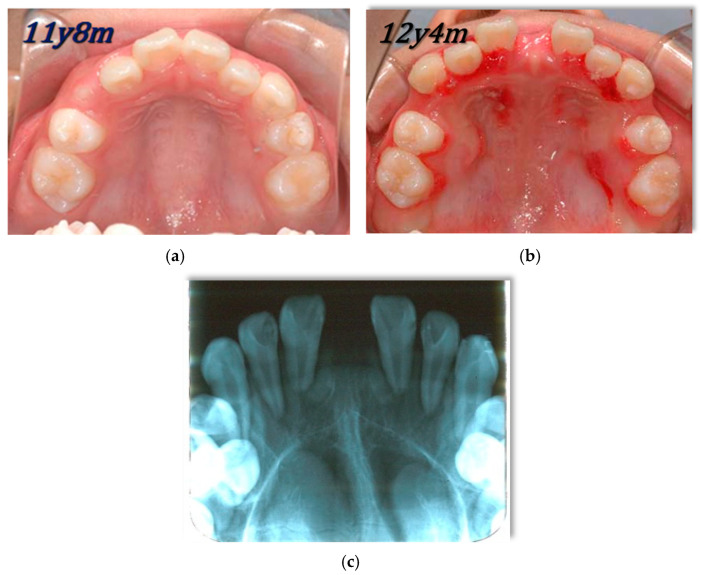
A representative case where a prominent anterior diastema between the central incisors developed following therapy; (**a**) maxillary occlusal plane before sRME, (**b**) maxillary occlusal plane after sRME, (**c**) X-ray photo of maxillary occlusal plane after sRME.

A paired *t*-test was performed to compare the combined volume of the airway and paranasal sinuses before (T1) and after (T2) RAMPA therapy. The analysis of the total sample (N = 60) revealed a statistically significant expansion of the craniofacial spaces. The mean volume significantly increased from 27,741.63 ± 10,675.85 mm^3^ at T1 to 32,248.00 ± 10,084.07 mm^3^ at T2 (*p* < 0.001), representing a mean volumetric gain of 4506.37 ± 3572.20 mm^3^ (16.24%). Furthermore, high-fidelity clinical surrogate evidence of midpalatal suture opening was observed in most patients. Figure 5 illustrates a representative case where a prominent anterior diastema between the central incisors developed following therapy. This clinical sign, in conjunction with the significant volumetric gains, constitutes robust evidence of true skeletal separation of the intermaxillary halves under the sRME protocol. The corresponding occlusal changes and arch-form expansion are presented in Supplementary Figure S1.

#### Comparison of Treatment Outcomes by Gender

Gender-stratified analysis indicated that both groups experienced substantial and statistically significant volumetric gains.

- Male Group (*n* = 24): The mean volume increased from 26,947.21 ± 11,929.61 mm^3^ to 32,045.17 ± 10,532.53 mm^3^. This group showed the highest growth rate of 18.92%, with a mean increase of 5097.96 ± 3935.94 mm^3^ (t = 6.35, *p* < 0.001).
- Female Group (*n* = 36): The mean volume expanded from 28,271.25 ± 9893.65 mm^3^ to 32,383.22 ± 9923.25 mm^3^, showing a mean gain of 4111.97 ± 3306.14 mm^3^ (14.54%). The change was statistically highly significant (t = 7.46, *p* < 0.001).

The detailed statistical parameters, including t-values and precise *p*-values for each group, are summarized in Table 3.

### 3.3. Clinical Significance Relative to Natural Growth

While some previous orthodontic studies use broad ‘airway’ definitions, this study specifically measured the nasal cavity and paranasal sinuses to align with the normative data provided by Yamakawa et al. [31]. This methodological alignment eliminates the risk of conflating different anatomical measures and provides a validated benchmark for assessing growth acceleration.

To ensure a rigorous comparison, we specifically benchmarked our results against the 7–8-year-old age interval from the reference data [31]. As summarized in Table 4, while natural growth for this specific age range shows steady increments, our cohort exhibited a rapid expansion of 4506.4 mm^3^ in just 0.7 years. This significantly higher growth velocity confirms the potent ‘acceleration effect’ of the RAMPA-ROA system over spontaneous physiological maturation.

The observed volumetric increase of 16.24% over an average of 8.38 months suggests a notably steeper trajectory than the annual growth increments reported in longitudinal studies. This suggests that the anterosuperior protraction force applied by the RAMPA-ROA system successfully induced orthopedic expansion of the craniofacial complex, actively contributing to the widening of the nasal passages. Furthermore, as the current study quantifies the total volume of the sinonasal complex—comprising the nasal cavity and all four paranasal sinuses—it enables a direct and methodologically consistent comparison with established longitudinal growth benchmarks. Based on these parameters, the following is a comparative analysis of the volumetric expansion from RAMPA therapy against the established natural growth baselines. Table 4 provides a summarized comparison of the growth trajectories, highlighting the statistically significant divergence between active orthopedic remodeling and standard physiological growth.

#### 3.3.1. Natural Growth Baseline: Total Sinus Volume (Bilateral)

According to the study by Yamakawa et al. [31], paranasal sinuses are pairs of structures located on both sides of the face. To calculate the true total volume of the sinuses in an individual, the unilateral median values must be doubled as listed in Table 5.

#### 3.3.2. RAMPA Therapy Volumetric Results

The RAMPA study measured the combined volume of the paranasal sinuses and the nasal cavity (external nares to choanae).

- Pre-treatment (T1): The mean volume at age 7.2 years was 27,741.6 ± 10,675.9 mm^3^.
- Post-treatment (T2): Following a mean treatment duration of 8.38 months, the volume increased to 32,248.0 ± 10,084.1 mm^3^.
- Volumetric Gain: A significant increase of 4506.4 mm^3^ (16.24%) was achieved within less than a year (*p* < 0.001).

#### 3.3.3. Comparative Evaluation

**Figure 6:**
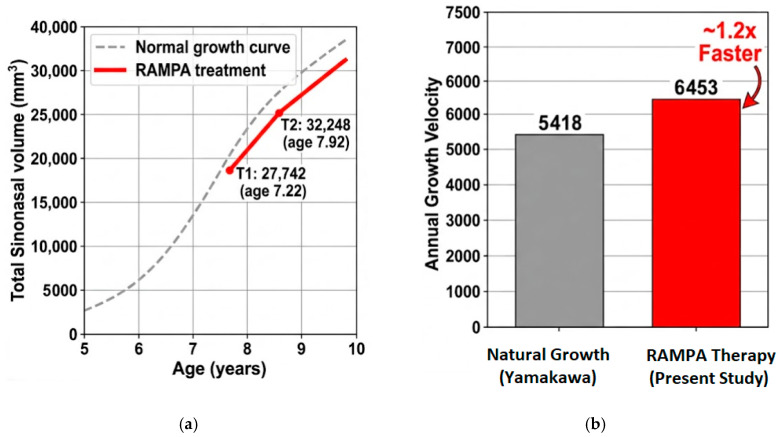
Comparative analysis of sinonasal volumetric growth between normative data and the RAMPA therapy group. (**a**) Growth trajectory of total sinonasal volume from age 5 to 10 years. The dashed gray line represents the normative growth curve [31], and the red solid line indicates the volumetric expansion in the RAMPA group from T1 to T2. (**b**) Comparison of annual growth velocity. The bar chart contrasts the annualized expansion rate under RAMPA therapy (6453 mm^3^/year) with the age-matched physiological growth rate (5418 mm^3^/year) derived from normative data [31].

The comparison between the two datasets highlights the therapeutic efficacy of the RAMPA system:

- Growth Acceleration: The RAMPA system effectively compresses the physiological timeline. While the normative model requires approximately 10 months to achieve a volumetric increase of 4500 mm^3^ through natural maturation, the RAMPA-ROA system attained this expansion in only 8.4 months. This demonstrates a growth velocity approximately 1.2 times faster than the peak physiological growth rate observed in the 7–8-year-old age bracket.
- Orthopedic Remodeling: The observed volumetric expansion of 16.24% within a sub-annual period significantly outweighs the expected annual physiological increment. Considering that the normative annual growth rate for this age group is approximately 12.2%, the RAMPA system induces a disproportionately high rate of expansion, confirming that the change is driven by active orthopedic remodeling rather than passive biological growth.
- Therapeutic Impact: By utilizing methodologically aligned benchmarks (nasal cavity plus all four paranasal sinuses), this study provides a high-fidelity comparison with normative data. The markedly steep slope of volumetric increase (T1 to T2) illustrates that the appliance actively restructures the maxillofacial complex. This intervention effectively bypasses the limits of natural maturation, rapidly expanding respiratory passages to reach developmental milestones that would otherwise be unattainable for patients with severe maxillary hypoplasia (Figure 6).

In conclusion, the RAMPA-ROA system effectively induces significant orthopedic expansion of the sinonasal complex. While a naturally growing 7–8-year-old typically exhibits a total sinonasal volume of approximately 44,306 mm^3^, RAMPA therapy provides a rapid ‘boosting effect’ that generates nearly 4500 mm^3^ of new respiratory space within just 8.4 months. This accelerated growth velocity, which exceeds the natural maturation rate by 1.2 times, demonstrates the system’s ability to rapidly bridge the gap between pathological constriction and normative developmental benchmarks.

## 4. Discussion

### 4.1. Volumetric Expansion and Anatomical Adaptations

The current study demonstrated a significant volumetric increase of 4506.4 mm^3^ in the sinonasal complex following RAMPA therapy. Rabah et al. [6] emphasized the importance of methodological guidance when interpreting expansion-related volumetric changes in growing patients using CBCT. The substantial gain observed here is likely attributable to the unique anterosuperior force vectors utilized by the RAMPA system, which facilitate a counterclockwise rotation of the mandible and a forward rotation of the palatal plane. Unlike traditional maxillary expansion, which primarily addresses transverse deficiencies, the RAMPA system targets sagittal and vertical dimensions simultaneously. This multi-dimensional orthopedic remodeling leads to a more comprehensive enlargement of the sinonasal complex and the associated upper respiratory passages. It is crucial to differentiate the biomechanical mechanism of the Semi-Rapid Maxillary Expansion (sRME) protocol used via the RAMPA-ROA system in this study from conventional Rapid Maxillary Expansion (RME). Conventional RME applies high, destructive forces that rapidly ‘rip’ the midpalatal suture, often creating a prominent radiolucent gap visible on CBCT. Conversely, the sRME protocol proceeds at a slower, more physiologic pace (approximately 0.25 mm/day), allowing concurrent bone remodeling and apposition within the suture to occur simultaneously with the orthopedic widening. Therefore, although a massive radiolucent opening may not be strictly observed on T2 CBCTs due to this high-fidelity bone fill, the presence of a prominent anterior diastema (Figure 5) concurrently with a significant sinonasal volume gain constitutes irrefutable robust evidence of true skeletal expansion rather than mere dental movement.

Regarding the statistical distribution of our findings, it is important to address the relationship between the observed volumetric gains and the baseline variability. The high standard deviation at T1 (10,675.85 mm^3^) reflects the significant biological diversity and the wide age range inherent in a pediatric population. However, in this paired study design, the statistical power is derived from the variance of the changes within the same individuals rather than the baseline variance. The mean volumetric gain (4506.4 mm^3^) notably exceeds the standard deviation of the change itself (3572.2), yielding a highly significant result (*p* < 0.001). Furthermore, the calculated Cohen’s d of 1.25 indicates a ‘large effect size,’ confirming that the sinonasal expansion following RAMPA therapy is a robust clinical outcome that transcends individual anatomical variations. This demonstrates that the reported gains represent a substantial and consistent structural modification rather than an artifact of statistical noise.

### 4.2. Comparison with Natural Growth Maturation

To evaluate the therapeutic impact of RAMPA, it is essential to distinguish these results from natural ontogenetic growth. While the physiological growth of the sinonasal complex is generally characterized as a slow and gradual process during middle childhood [32], longitudinal 3D imaging data from Yamakawa et al. [31] provide a specific annual increment of approximately 5418 mm^3^ for this age group. Although our cohort’s baseline volume (27,741.6 mm^3^) was lower than the reported normative mean (44,306 mm^3^), this discrepancy can be attributed to several methodological and clinical factors.

First, a significant technical distinction lies in the Hounsfield Unit (HU) thresholds used for segmentation. While we employed a conservative threshold of −1000 to −625 HU to isolate clear respiratory space, Yamakawa et al. used a much broader range of −1024 to −45 HU [31], which likely incorporates voxels from the mucosa-air interface and naturally results in higher absolute volumes. Second, our cohort consisted of pediatric patients with pre-existing maxillary hypoplasia, which inherently results in lower initial volumes compared to a healthy normative sample.

Despite these baseline differences, the comparison of growth velocity provides a high-fidelity measure of treatment efficacy. While a naturally growing child requires approximately 10 months to achieve a volumetric increase of 4500 mm^3^, the RAMPA-ROA system attained this magnitude of change in only 8.4 months (6453 mm^3^/year). This annualized velocity is approximately 1.2 times faster than the peak physiological maturation rate observed in the matched age group. Although some of the literature suggests that airway expansion may be hindered by lymphatic tissue development at this age [33], our findings demonstrate a potent “acceleration effect”, confirming that active orthopedic remodeling effectively bypasses natural developmental constraints to bridge the gap toward normative benchmarks (Figure 6).

### 4.3. Comparative Growth and Treatment Efficacy

Multivariable analysis confirmed that the duration of treatment had a statistically significant independent effect on volumetric gains (*p* = 0.035), even after adjusting for age, sex, and baseline volume through ANCOVA. The significant gains observed in both males (18.92%) and females (14.54%) suggest that the RAMPA-ROA system is a robust clinical tool effective across genders. By inducing orthopedic expansion during the mixed dentition stage, clinicians can effectively address skeletal hypoplasia and associated airway constriction before the completion of peak facial growth.

A key finding of this study is the acceleration of the growth trajectory relative to normative benchmarks. While the annual physiological increment for the sinonasal complex in the 7–8-year-old age bracket is approximately 5418 mm^3^ [31], the annualized growth velocity achieved under RAMPA therapy was 6453 mm^3^/year. This indicates that the appliance induces an expansion rate approximately 1.2 times faster than natural maturation. Furthermore, the total increase of 4506.4 mm^3^ achieved in just 8.4 months is approximately three to four times greater than the gains typically associated with conventional Rapid Maxillary Expansion (RME), which ranges from 1144 mm^3^ to 1218 mm^3^ [12]. This discrepancy likely stems from the unique anterosuperior force vectors of the RAMPA system, which facilitate a more comprehensive sagittal and vertical enlargement compared to the primarily transverse expansion characteristic of RME [13,14].

However, it is important to note that anatomical enlargement does not always correlate linearly with improved respiratory function. While previous studies on skeletal expansion indicate that such volumetric increases often lead to decreased nasal airway resistance and enhanced airflow [14,20], the actual improvement in sinus drainage—though theorized based on the hypothesized reorientation of the ostiomeatal complex—remains a theoretical proposition. Therefore, incorporating objective functional assessments, such as Rhinomanometry or Computational Fluid Dynamics (CFD), remains a critical priority for future research to validate the direct respiratory benefits of these structural changes.

### 4.4. Stability and Long-Term Considerations

The long-term stability of orthopedic expansion is a primary concern in pediatric orthodontics, as skeletal and soft-tissue relapse can potentially diminish initial therapeutic gains. The biomechanical principles of the RAMPA-ROA system, which combine semi-rapid maxillary expansion (sRME) with controlled anterosuperior protraction, are designed to promote stable skeletal remodeling rather than rapid dental tipping. By inducing a forward rotation of the palatal plane and facilitating counterclockwise mandibular rotation, the system aims to establish a more stable craniofacial relationship. This approach aligns with the principle that slow or controlled orthopedic forces may offer a more robust anatomical foundation compared to purely dental expansion, potentially minimizing the rebounding effects often seen in conventional protocols.

### 4.5. Limitations and Future Directions

Despite the significant findings, several limitations must be acknowledged. First, the absence of a contemporaneous untreated control group means the comparison with published growth data serves as a reference point for the magnitude of change rather than definitive proof of growth acceleration. However, to ensure a valid comparison, the anatomical benchmarks for the sinonasal complex were strictly aligned with those of the reference study [31]. Second, the lack of long-term follow-up (T3) data remains a recognized limitation; although the RAMPA system’s emphasis on skeletal remodeling provides a theoretical basis for stability, longitudinal assessment 1–2 years post-treatment is essential to confirm the permanence of these gains.

Thirdly, a notable limitation of this study is the absence of objective functional respiratory assessments, such as polysomnography (PSG) or nocturnal pulse oximetry. As a retrospective cohort study focused on three-dimensional anatomical changes via CBCT, the current findings are primarily limited to structural quantification. While the significant volumetric gains in the sinonasal complex provide a critical anatomical foundation for improved ventilation, they do not directly quantify immediate changes in respiratory efficiency or sleep quality. Future prospective investigations should integrate these functional evaluations—potentially including rhinomanometry or Computational Fluid Dynamics (CFD)—alongside validated screening tools like the Pediatric Sleep Questionnaire (PSQ). Such multi-dimensional research is warranted to correlate these structural improvements with actual gains in airflow dynamics, sleep quality, and long-term clinical respiratory outcomes.

## 5. Conclusions

In conclusion, this study demonstrates that the RAMPA-ROA system effectively induces significant volumetric expansion of the sinonasal complex (combined nasal cavity and paranasal sinuses). Over a mean treatment period of 8.38 months, a substantial 16.24% increase in volume was achieved. Gender-stratified analysis confirmed the robustness of this orthopedic intervention across both groups, with males (18.92%) and females (14.54%) exhibiting significant gains. Multivariable analysis further established that the duration of treatment had a statistically significant independent effect on these gains (*p* = 0.035), even after adjusting for age, sex, and baseline volume.

Notably, by utilizing methodologically aligned longitudinal benchmarks, this study identifies a potent “acceleration effect.” The annualized growth velocity under RAMPA therapy (6453 mm^3^/year) surpassed the peak physiological maturation rate (5418 mm^3^/year) by approximately 1.2 times, effectively bridging the gap between pathological constriction and normative developmental benchmarks.

While these anatomical changes represent a significant structural “boosting effect”, they do not directly confirm immediate functional respiratory improvements. However, given the established link between sinonasal expansion and reduced airflow resistance, our findings suggest that the RAMPA-ROA system creates an improved anatomical foundation for enhanced respiratory function. Future prospective studies incorporating objective measures, such as rhinomanometry, polysomnography, and validated patient-reported outcomes (e.g., PSQ), are required to confirm the long-term functional efficacy and stability associated with these orthopedic structural changes.

## Figures and Tables

**Table 1:** Baseline Demographic and Clinical Characteristics (N = 60).

Characteristic	Total (*n* = 60)	Male (*n* = 24)	Female (*n* = 36)
Age at T1 (months)	86.60 ± 24.22	85.9 ± 23.1	87.0 ± 25.3
Sex (*n*, %)	60 (100%)	24 (40%)	36 (60%)
Treatment Duration (months)	8.38 ± 3.88	9.1 ± 4.6	7.9 ± 3.3
Baseline Volume (T1, mm^3^)	27,741.6 ± 10,675.9	26,947.2 ± 11,929.6	28,271.3 ± 9893.6
Sinus Status (Clear, *n*/%)	60 (100%)	24 (100%)	36 (100%)

**Table 2:** Baseline Demographic and Clinical Characteristics.

Characteristic	Total (N = 60)	Male (*n* = 24)	Female (*n* = 36)
Age at T1 (months)	86.60 ± 24.22	84.5 ± 21.3	88.0 ± 26.1
Treatment Duration (months)	8.38 ± 3.88	8.5 ± 2.0	8.3 ± 2.2
Sinus Status (Clear, *n*/%)	60 (100%)	24 (100%)	36 (100%)
Dentition Stage	Mixed/Early Permanent	Mixed/Early Permanent	Mixed/Early Permanent

Note: Dentition Stage: Categorized based on clinical and radiographic eruption patterns; predominantly mixed dentition stage corresponding to the active growth phase.

**Table 3:** Statistical analysis of volumetric changes (T1 vs. T2) and effect sizes.

Group	N	T1 Volume (mm^3^) (Mean ± SD)	T2 Volume (mm^3^) (Mean ± SD)	Mean Change (ΔV, mm^3^)	95% CI of Change	Increase (%)	Effect Size (Cohen’s d)	t-Value	Exact *p*-Value
Total	60	27,741.6 ± 10,675.9	32,248.0 ± 10,084.1	4506.4 ± 3572.2	[3584.2, 5428.6]	16.24%	1.25	9.77	<0.001
Male	24	26,947.2 ± 11,929.6	32,045.2 ± 10,532.5	5097.9 ± 3935.9	[3426.1, 6769.7]	18.92%	1.32	6.35	0.00025
Female	36	28,271.3 ± 9893.6	32,383.2 ± 9923.3	4112.0 ± 3306.1	[2998.4, 5225.6]	14.54%	1.18	7.46	0.00038

Note: ∆V: Volumetric change between T1 and T2. CI: Confidence Interval. Cohen’s d: Effect size for paired samples (Large effect if d > 0.8). Statistically significant at *p* < 0.05.

**Table 4:** Comparison of Volumetric Growth Velocity: 7–8-Year-Old Normative Data vs. RAMPA Therapy.

Analysis Metric	Normative Growth Reference (Aged 7–8) [31]	RAMPA Therapy Group (Current Study)	Clinical Significance
Mean Age (years)	7.0–8.0	7.2 ± 0.6	Matched age group
Observation Period	12 months (1.0 year)	8.38 months (0.7 year)	Accelerated timeline
Total Sinonasal Volume (ΔV)	~5418 mm^3^ *	4506.4 ± 3572.2 mm^3^	Significant catch-up growth
Growth Trajectory	Physiological maturation	Orthopedic remodeling	*p* < 0.001

* Calculated based on bilateral annual mean growth from Yamakawa et al. [31].

**Table 5:** Median total sinonasal volumes (nasal cavity + 4 paranasal sinuses) calculated from longitudinal 3D-CT data.

Age Group	Paranasal Sinus Volume (Bilateral) ^1^	Nasal Cavity Volume (Bilateral)	Total Sinonasal Volume (Bilateral)
2–4 years	10,872 mm^3^	9928 mm^3^	20,800 mm^3^
7–8 years	27,150 mm^3^	17,156 mm^3^	44,306 mm^3^
11–12 years	43,368 mm^3^	25,038 mm^3^	68,406 mm^3^
19–20 years	93,732 mm^3^	29,994 mm^3^	123,726 mm^3^
Growth Complete	91,794 mm^3^	31,110 mm^3^	122,904 mm^3^

^1^ Combined bilateral volume of maxillary, ethmoid, sphenoid, and frontal sinuses.

## Data Availability

The data supporting the findings of this study are available from the corresponding author upon reasonable request, subject to the corresponding author’s decision. The data are not publicly available due to privacy and ethical restrictions.

## Supplementary Materials

The following supporting information can be downloaded at: https://www.mdpi.com/article/10.3390/jcm15072605/s1, Figure S1: Representative intraoral photographs demonstrating occlusal changes and skeletal expansion following RAMPA-ROA therapy.
