# Supplementary material for: CBCT Volumetric Changes in Combined Nasal Cavity and Paranasal Sinuses Following RAMPA-ROA Therapy: A Retrospective Cohort Study with Reference to Longitudinal Growth Data
Source: J Clin Med. 2026 Mar 29;15(7):2605. doi: 10.3390/jcm15072605 (PMC13074101; doi:10.3390/jcm15072605)
Supplement: Supplementary file 1 [file jcm-15-02605-s001.zip › jcm-4148483-supplementary.pdf]

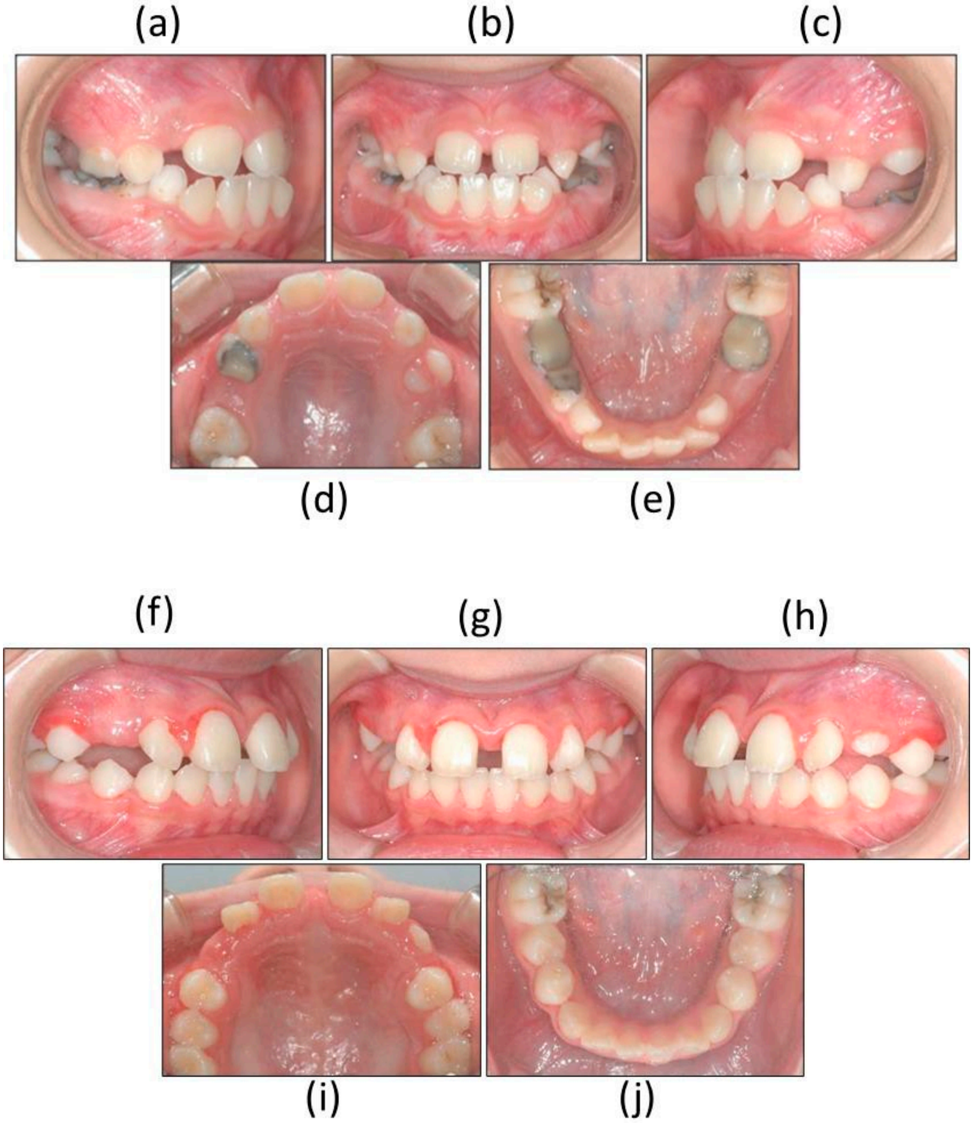

Supplementary Figure S1. Representative intraoral photographs demonstrating occlusal changes and skeletal expansion following RAMPA-ROA therapy.

Upper Row (T1: Pre-treatment): (a) Left 45-degree oblique view, (b) Frontal view of the occlusion, (c) Right 45-degree oblique view, (d) Maxillary occlusal view showing a constricted V-shaped arch, and (e) Mandibular occlusal view. Lower Row (T2: Post-treatment): (f) Left 45-degree oblique view, (g) Frontal view showing the development of an anterior diastema, (h) Right 45-degree oblique view, (i) Maxillary occlusal view illustrating the conversion to a broad U-shaped arch, and (j) Mandibular occlusal view.
